# Screening of Big Pharma’s Library against Various *in-house* Biological Targets

**DOI:** 10.3390/molecules27144484

**Published:** 2022-07-13

**Authors:** Damijan Knez, Stanislav Gobec, Martina Hrast

**Affiliations:** Faculty of Pharmacy, University of Ljubljana, Aškerčeva 7, SI 1000 Ljubljana, Slovenia; damijan.knez@ffa.uni-lj.si (D.K.); stanislav.gobec@ffa.uni-lj.si (S.G.)

**Keywords:** Merck Mini Library, screening, MurA, MurC, human MAO-A/B, human BChE, murine AchE

## Abstract

Open innovation initiatives provide opportunities for collaboration and sharing of knowledge and experience between industry, academia, and government institutions. Through open innovation, Merck is offering a Mini Library of 80 carefully selected compounds from previous research and development projects to a broader scientific community for testing in academic drug discovery projects. These compounds are predominantly drug-like and cover a broad range of molecular targets. They could potentially interact with other enzymes, receptors, transporters, and ion channels of interest. The Mini Library was tested on seven *in-house* enzymes (bacterial MurA, MurC ligase, and DdlB enzyme, human MAO-A/B, human BChE, and murine AChE), and several hits were identified. A follow-up series of structural analogues provided by Merck gave a more detailed insight into the accessibility and the quality of the hit compounds. For example, sartan derivatives were moderate inhibitors of MurC, whereas bisarylureas were potent, selective, nanomolar inhibitors of hMAO-B. Importantly, 3-*n*-butyl-substituted indoles were identified as low nanomolar selective inhibitors of hBChE. All in all, the hit derivatives provide new starting points for the further exploration of the chemical space of high-quality enzyme inhibitors.

## 1. Introduction

Pharmaceutical companies and academic research groups often begin their small molecule drug discovery programs by screening compound libraries against biological targets to identify hit compounds that are, subsequently, optimized for potency, physicochemical, and ADMET properties [1,2,3]. Roughly, more than 16 million compounds are available from commercial vendors [4], and large pharmaceutical companies also have internal screening libraries assembled from several million compounds from previous projects [5]. Despite the development of several alternative approaches to drug design, such as virtual screening [6], the fragment-based hit-to-lead design [7], DNA-encoded libraries [8], and others, high-throughput screening (HTS) remains the most effective method for discovering hits and leads [5]. HTS has served as the starting point for numerous approved drugs [9], such as sitagliptin (DPP IV inhibitor) and rivaroxaban (factor Xa inhibitor). However, two features are critical to the success of a compound library screening: a robust and sensitive biochemical assay against the essential biological target and the quality of the compound library [10]. The most important features that determine the quality of the screening library are size, structural diversity, novelty, purity, and pharmaceutical properties, i.e., lead and/or drug-like properties [5]. In addition, problematic compounds, such as frequent hitters, reactive moieties, or redox-cycling compounds, should be identified and eliminated [10,11].

Drug repurposing is a promising approach to rapidly find new therapeutic options for existing drugs [12]. Hits derived from approved drugs have a significant advantage in the drug optimization process, as a considerable amount of biological data is already available. As part of the open innovation initiative, Merck offers a curated Mini Library of compounds to a broader scientific community that can be tested in biochemical and cell-based assays to identify hits with novel and promising biological activities [13]. Therefore, the Mini Library was assayed against several *in-house* targets: bacterial MurA, MurC, and DdlB ligases, human butyrylcholinesterase (hBChE), murine acetylcholinesterase (mAChE), and human monoamine oxidases A and B (hMAO-A/B).

Briefly, MurA, MurC, and DdlB ligases are essential bacterial enzymes involved in the cytoplasmic steps of bacterial cell wall biosynthesis [14]. Despite the extensive knowledge of their enzyme kinetics, catalytic mechanisms, and available crystal structures, these enzymes are underutilized as antibacterial targets [15]. On the other hand, BChE, AChE, and MAO-A/B are well-validated targets for neurological disorders, such as Alzheimer’s disease, Parkinson’s disease, and depression, for which several drugs have already been approved [16,17,18,19]. However, recent findings from biological and longitudinal clinical studies are expanding the therapeutic applicability of ligands targeting the above enzymes to other neurodegenerative, cardiac, and malignant diseases [20,21,22,23,24]. The lack of effective ligands against bacterial enzymes and the emerging therapeutic options for the human enzymes described above prompt further drug discovery efforts to find new, well-characterized ligands with desired pharmacological activities.

## 2. Results and Discussion

Merck’s Mini Library is part of the open innovation initiative, and contains drug-like compounds from former Merck Biopharma research and development projects. Most of these compounds have been well-characterized in vitro and in vivo against their primary targets and toxicologically evaluated to rule out toxic compounds. In addition, clinical data, such as human pharmacokinetics and human safety, are available for certain ligands.

Initially, 80 compounds from Merck’s Mini Library were tested at a concentration of 100 µM against the bacterial enzymes MurA, MurC, and DdlB, and at a concentration of 10 µM against the remaining enzymes, i.e., hBChE, mAChE, hMAO-A, and hMAO-B (Supporting Information, Table S1). Results were expressed as residual activities (RAs) at the indicated compound concentration. Three compounds showed a moderate inhibition of MurA, three sartan derivatives inhibited MurC, four compounds were active against hBChE, three against mAChE, and only two inhibited hMAO-B. No active compounds were found against DdlB and hMAO-A. To determine the IC_50_ values and to explore the chemical space around the active ligands, Merck secured sufficient amounts of active compounds and their structural analogues.

**Figure 1:**
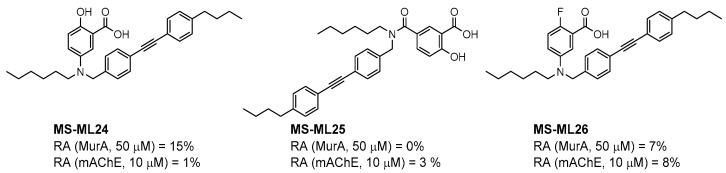
Structures and in vitro inhibitory potencies of hits from class of salicylic acid analogues.

Salicylic acid analogues have been described as potent and selective inhibitors of glomerular epithelial protein-1 [25], which plays an important role in controlling the chemotaxis of various types of leukocytes. In our screening campaign, they were identified as inhibitors of the bacterial enzymes MurA, mAChE, and hBChE (Figure 1, Table 1, and Table S2). In general, the compounds inhibited mAChE in the nanomolar range, and MurA was inhibited with IC_50_ values in the low micromolar range. The substitution of the hydroxyl group with fluorine did not affect the inhibitory potency on both MurA and mAChE (**MS-ML24** vs. **MS-ML26**). The introduction of an amide bond also did not alter the inhibitory potencies to a significant extent (**MS-ML24** vs. **MS-ML25**). The preferred substitution at the diphenylacetylene moiety was a linear alkyl chain, i.e., *n*-butyl, while the alkyl substituent at the amine/amide did not affect the observed activities. Unfortunately, these compounds did not show clear structure–activity relationships (SARs) against any of the targets, which was also reflected in higher Hill coefficients (>1.5), which could also indicate a nonspecific inhibition due to the highly lipophilic chemical structure, leading to poor solubility in the micromolar concentration range [26]. In addition, the presence of negatively charged moieties—i.e., carboxylic acids—is not optimal for permeation through the blood–brain barrier, which is a prerequisite for target enzymes located in the central nervous system (i.e., AChE and BChE), making salicylic acid analogues of no major interest for further development as cholinesterase inhibitors [27].

4,5-Dihydro-4-oxo-3*H*-imidazo[4,5-*c*]pyridine derivatives are nonpeptide angiotensin II receptor antagonists. This class of compounds, named sartans, is widely used in clinics for the treatment of hypertension and heart failure [28,29,30]. The follow-up sartan derivatives were assayed at 100 µM against MurC, and the IC_50_ values were determined for compounds showing RAs below 50% (Table 2). Most sartan analogues inhibited MurC in a micromolar concentration range, with IC_50_ values of approximately 100 µM. Variously substituted benzyls were well-tolerated at position five of the 3,5-dihydro-4*H*-imidazo[4,5-*c*]pyridin-4-one core, whereas the introduction of the acyclic *tert*-butyl moiety was not tolerated and resulted in the loss of MurC inhibition (compounds **15** and **17**). To further explore the SARs of the sartan analogues against MurC, a larger number of compounds would need to be tested.

The Mini Library screening identified two structurally distinct classes of hMAO-B inhibitors (Table 3 and Table S3). Heterocyclic-substituted bisarylureas were patented by Merck as kinase inhibitors; notably, these compounds inhibit the VEGF-stimulated mitogenesis of human vascular endothelial cells [31]. In addition, triazaindolizines have been patented as inhibitors of methionine aminopeptidase 2 [32]. Several bisarylureas inhibited hMAO-B in the low micromolar to submicromolar range, and small structural changes resulted in large differences in inhibitory potencies. For example, the introduction of *N*-methylacetamide on the pyridine ring resulted in the complete loss of activity, whereas the replacement of the pyridine moiety with aminopurine (**22**) or the complete removal of the pyridine to obtain *p*-aniline analogue **23** resulted in equipotent inhibitors. The introduction of substituents on the pyridine ring, e.g., *N*,*N*-dimethylethyl (**27**) or piperidine-1-ethyl (**28**), resulted in active compounds. Only triazaindolizine **MS-ML31** with the phenyloxypyridinyl fragment inhibited hMAO-B (Table 3). The replacement of phenyloxypyridine with substituted quinolines, (tetrahydro)isoquinolines, and phenyls completely abolished hMAO-B inhibition (Table S3). All compounds were inactive against hMAO-A, indicating that bisarylureas were selective hMAO-B inhibitors.

A number of indoles were described by Merck as dopaminergic receptor agonists [33], and these compounds were found to be potent hBChE inhibitors (Table 4). The most potent inhibitors were analogues with unsubstituted or substituted indoles via an *n*-butyl linker to 4-phenyl-1,2,3,6-tetrahydropyridine. Bulky groups such as naphthene (**30**) or 6-methoxy-2-methyl-2,3,4,9-tetrahydro-1*H*-pyrido[3,4-*b*]indole (**37**) linked to 1,2,3,6-tetrahydropyridine reduced hBChE inhibition. All compounds were also assayed for their mAChE inhibition, and most of them were selective hBChE inhibitors. Indole and tryptophan derivatives are known hBChE inhibitors and have also been extensively studied in our group as promising pharmacological tool molecules and lead compounds for the treatment of Alzheimer’s disease [34,35].

## 3. Materials and Methods

Inhibitory activities were expressed as residual activities (RAs) in percent. IC_50_ values were determined for the most potent inhibitors and were given as IC_50_ ± SEM. Compounds were provided by Merck as 10 mM stock solutions in DMSO.

### 3.1. Inhibition of Mur Enzymes and DdlB

Inhibition of Mur enzymes was monitored with the colorimetric malachite green method, which measured orthophosphate formed during reaction [36]. The mixtures with a final volume of 50 µL contained:

MurA: 50 mM HEPES-NaOH, pH 7.8, 0.005% Triton X-114, 200 µM uridine-diphosphate-*N*-acetylglucosamine, 100 µM phosphoenolpyruvate, purified MurA, and 50 or 100 µM of each compound tested.

MurC: 50 mM HEPES-NaOH, pH 8.0, 5 mM MgCl_2_, 0.005% Triton X-114, 120 µM L-Ala, 120 µM UDP-*N*-acetyl-muramic acid, 450 µM ATP, purified MurC, and 50 or 100 µM of each compound tested.

DdlB: 50 mM HEPES-NaOH, pH 8.0, 5 mM MgCl_2_, 6.5 mM (NH_4_)_2_SO_4_, 10 mM KCl, 0.005% Triton X-114, 700 µM D-Ala, 500 µM ATP, purified DdlB, and 50 µM of each compound tested.

All compounds were soluble in the assay mixtures containing 5% DMSO (*v*/*v*). After incubation for 15 min (20 min for DdlB) at 37 °C, the enzyme reaction was terminated by addition of Biomol^®^ reagent (100 µL) and absorbance was measured after 5 min at 650 nm (Synergy™ H4 microplate reader; BioTek Instruments, Inc., BioTek Gen5 Data Analysis Software, version 2.9, Santa Clara, CA, USA). All experiments were performed in duplicate. Residual activities (RAs) were calculated in comparison to assays, where 5% DMSO replaced the compound, and IC_50_ values were determined by measuring the RAs at seven different compound concentrations.

### 3.2. Inhibition of hBChE and mAChE

Enzyme solutions were prepared by diluting the concentrated enzyme stock solutions (4.6 mg/mL in 10 mM MES-NaOH buffer, pH 6.5) in a sodium phosphate-buffered solution (0.1 M, pH 8.0). Reactions were performed in 0.1 M phosphate-buffered solution, pH 8.0, containing 370 μM 5,5′-dithiobis(2-nitrobenzoic acid) (DTNB), 500 μM substrate (butyrylthiocholine and acetylthiocholine for hBChE and mAChE, respectively), and 1 nM hBChE or 50 pM mAChE. The final organic solvent (DMSO) content was always 1% (*v*/*v*). Reactions were initiated by adding the substrate to the enzyme and inhibitor, which were preincubated for 5 min. The increase in absorbance at 412 nm was monitored for 2 min using a microplate reader (Synergy™ H4 microplate reader; BioTek Instruments, Inc., BioTek Gen5 Data Analysis Software, version 2.9, Santa Clara, CA, USA). To determine the blank value (*b*), the enzyme solution was replaced by a phosphate-buffered solution. The initial velocity (*v*) was calculated from the slope of the linear trend obtained, with each measurement performed in triplicate. The inhibitory potencies were expressed as RA=(vi − b)/(vo − b), where *v_i_* is the velocity in the presence of the test compounds and *v_o_* is the control velocity in the presence of DMSO. Seven different concentrations of each compound were used for the IC_50_ measurements. IC_50_ values were obtained by plotting the residual enzyme activities against the applied inhibitor concentrations, fitting the experimental data to the 4-parameter Hill equation:

(1) $$Y=\frac{Bottom+\left(Top-Bottom\right)}{1+10^{\begin{array}{c} \left(\left(LogIC50-X\right)\times Hill\;Slope\right) \end{array}}}$$

where *X* is the logarithm of the inhibitor concentration, and *Y* is the residual activity. GraphPad Prism 8.2.0 (GraphPad Software, San Diego, CA, USA) was used for the fitting procedure.

### 3.3. Inhibition of hMAO-A and hMAO-B

The effects of the compounds on hMAO-A and hMAO-B were investigated using a fluorimetric assay, following a method previously described in the literature [37]. Recombinant human microsomal hMAO enzymes expressed in BTI-TN-5B1-4 baculovirus-infected insect cells, horseradish peroxidase (HRP, type II, lyophilized powder), and *p*-tyramine hydrochloride were purchased from Sigma Aldrich. 10-Acetyl-3,7-dihydroxyphenoxazine (Amplex Red reagent) was synthesized as described in the literature [38].

Briefly, 100 µL of 50 mM sodium phosphate buffer (pH 7.4, 0.05% (*v*/*v*) Triton X-114), containing the compounds and hMAO-A or hMAO-B, was incubated for 15 min at 37 °C in a flat-bottomed black 96-well microplate protected from ambient light. After preincubation, the reaction was started by adding final concentrations of 200 µM Amplex Red reagent, 2 U/mL HRP, and 1 mM *p*-tyramine hydrochloride. Resorufin production was quantified based on the fluorescence produced (λex = 530 nm; λem = 590 nm) at 37 °C over a 20 min period. For the control experiments, DMSO was used instead of appropriate dilutions of the compounds in DMSO. For the determination of blank (*b*), the enzyme solution was replaced by a phosphate-buffered solution. Initial velocities were calculated from the obtained trend, with each measurement performed in duplicate. Specific fluorescence emission to determine the final results was calculated after subtracting the blank activity (*b*). Inhibitory potencies were expressed as RAs, and IC_50_ values were calculated as described in the section “Inhibition of hBChE and mAChE” above.

## 4. Conclusions

Overall, compound library screening is a viable route to obtaining novel ligands targeting underexploited proteins in the field of antibacterial agents or fully validated enzymes that form the core of Alzheimer’s and Parkinson’s disease therapy. We performed a successful screening of Merck’s Mini Library on seven *in-house* enzymes, revealing several hit compounds. Particular attention should be paid to validate the activity detected in the screening phase and not to pursue hits with questionable activity. Salicylic acid analogues fell into the latter category, while sartans, as moderate MurC inhibitors, needed to be validated in secondary assays to confirm the clear SARs of these hits. On the other hand, bisarylureas and indoles as selective, nanomolar hMAO-B and hBChE inhibitors, respectively, in combination with Merck’s data on these derivatives (e.g., selectivity, preclinical pharmacokinetics, and toxicology) [39,40,41,42,43,44,45,46,47,48,49], provided a solid foundation for the further exploration of their activities. Further cell-based and in vivo rodent models of neurodegenerative diseases should be used to reveal the true potential of hit compounds as potential agents against neurodegenerative disorders.

## Figures and Tables

**Table 1:** In vitro inhibitory potencies of salicylic acid analogues against MurA, mAChE, and hBChE.

Compound	MurA		mAChE		hBChE	
	RA ^a^ (%)	IC_50_ (µM)	RA ^b^ (%)	IC_50_ (nM)	RA ^b^ (%)	IC_50_ (nM)
**MS-ML24**	0	7 ± 3	1	294 ± 25	9	949 ± 46
**MS-ML25**	0	6 ± 2	3	485 ± 79	55	/
**MS-ML26**	0	7 ± 4	8	275 ± 60	57	/
**1**	0	No clear dose-dependency	2	1200 ± 50	100	/
**2**	64	/	99	/	100	/
**3**	58	/	100	/	100	/
**4**	14	102 ± 15	100	/	100	/
**5**	0	7 ± 3	2	1100 ± 100	16	2400 ± 200
**6**	0	27 ± 7	13	4800 ± 1300	95	/
**7**	100	/	19	682 ± 86	45	19,700 ± 10,000 ^c^
**8**	0	14 ± 5	22	368 ± 52	71	/
**9**	10	91 ± 13	52	1800 ± 450	80	/
**Fosfomycin**	0	0.24 ± 0.02	/	/	/	/
**Tacrine**			0	106 ± 10	0	12 ± 3

^a^ RA was determined at 100 µM; ^b^ RA was determined at 10 µM. ^c^ Poor solubility, estimated IC_50_ value.

**Table 2:**
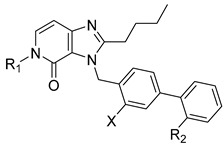
In vitro inhibitory potencies of sartan derivatives against MurC.

Compound	R_1_	R_2_	X	MurC	
				RA ^a^ (%)	IC_50_ (µM)
**MS-ML03**	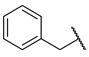	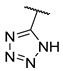	H	40	85 ± 15
**MS-ML05**	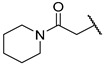	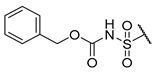	F	32	101 ± 23
**10**	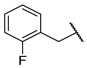	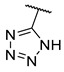	H	30	92 ± 11
**11**	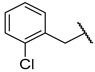	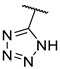	H	20	77 ± 12
**12**	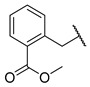	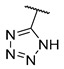	H	27	84 ±15
**13**	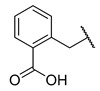	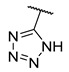	H	34	109 ± 26
**14**	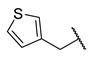	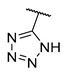	H	26	90 ± 13
**15**	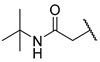	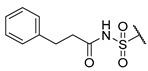	F	59	/
**16**	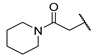	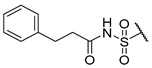	F	53	/
**17**	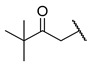	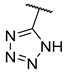	H	75	/
**18**	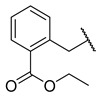	−CN	H	58	/
**19**	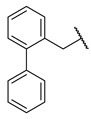	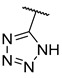	H	15	75 ± 9

^a^ RA was determined at 100 µM.

**Table 3:**
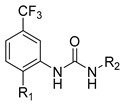
In vitro inhibitory potencies of bisarylureas against hMAO-A and hMAO-B, and representative triazaindolizine inhibitor of hMAO-B.

Compound	R_1_	R_2_	hMAO-B		hMAO-A
			RA ^a^ (%)	IC_50_ (nM)	RA ^a^ (%)
**MS-ML80**	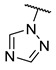	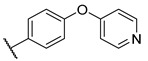	10	405 ± 76	78
**20**	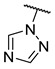	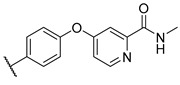	99	/	60
**21**	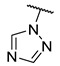	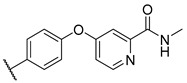	88	/	92
**22**	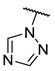	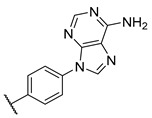	6	710 ± 100	111
**23**	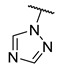	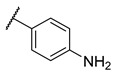	24	3000 ± 800	100
**24**	–F	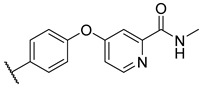	100	/	100
**25**	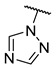	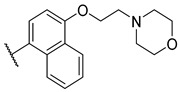	86	/	79
**26**	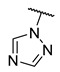	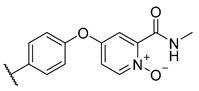	98	/	89
**27**	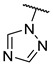	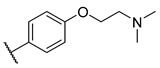	6	465 ± 42	117
**28**	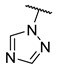	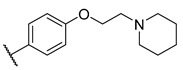	15	2600 ± 100	94
**29**	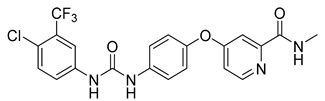		89	/	86
**Triazaindolizine**					
**MS-ML31**	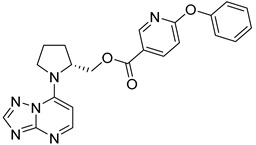		17	912 ± 122	98
**Safinamide**			0	29 ± 2	/
**Harmaline**			/	/	0 ^b^

^a^ RA was determined at 10 µM; ^b^ IC_50_ = 32.9 ± 5.2 nM.

**Table 4:**
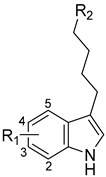
In vitro inhibitory potencies of indoles against hBChE and mAChE.

Compound	R_1_ *	R_2_	hBChE		mAChE	
			RA ^a^ (%)	IC_50_ (nM)	RA ^a^ (%)	IC_50_ (µM)
**MS-ML10**	–H	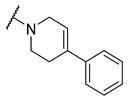	0	35.6 ± 0.3	64	/
**MS-ML11**	–OH (5)	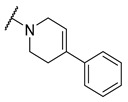	0	78.8 ± 10.3	82	/
**30**	–H	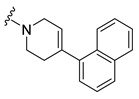	6	400 ± 57	76	/
**31**	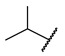 (5)	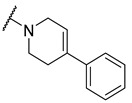	0	59.9 ± 5.5	44	14 ± 4
**32**	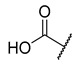 (4)	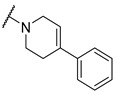	49	14500 ± 2200	71	/
**33**	–OH (6)	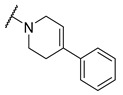	3	267 ± 1	61	/
**34**	–H	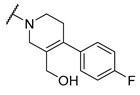	7	493 ± 88	31	7 ± 1
**35**	–CN (5)	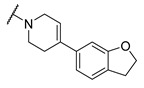	31	2900 ± 100	59	/
**36**	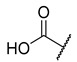 (5)	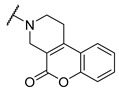	97	/	78	/
**37**	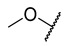 (5)	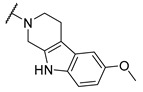	23	2300 ± 100	88	/
**38**	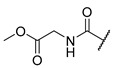 (5)	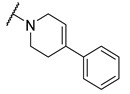	4	319 ± 51	100	/
**39**	–F (5)	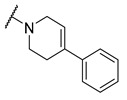	0	70 ± 5	47%	15 ± 5

^a^ RA was determined at 10 µM; * numbers represent the position of substituent on the indole ring.

## Data Availability

The data that support the findings of this study are available from the corresponding author, M.H., upon reasonable request.

## Supplementary Materials

The following supporting information can be downloaded at: https://www.mdpi.com/article/10.3390/molecules27144484/s1, Table S1: In vitro inhibitory potencies of Merck’s Mini Library against MurA, MurC, DdlB, mAChE, hBChE, hMAO-A/B; Table S2: Structures of salicylic acid derivatives; Table S3: In vitro inhibitory potencies of triazaindolizines derivatives against hMAO-B and hMAO-A.

## Sample Availability

Samples of the compounds are available from Merck upon request (https://www.merckgroup.com/en/research/open-innovation/biopharma-open-innovation-portal/biopharma-mini-library.html, accessed on 15 May 2022).
