# Supplementary material for: Screening of Big Pharma’s Library against Various in-house Biological Targets
Source: Molecules. 2022 Jul 13;27(14):4484. doi: 10.3390/molecules27144484 (PMC9320114; doi:10.3390/molecules27144484)
Supplement: Supplementary file 1 [file molecules-27-04484-s001.zip › molecules-1754325-SI.pdf]

SUPPORTING INFORMATION

Screening of Big Pharma's library against various *in-house* biological targets

Damijan Knez[a], Stanislav Gobec[a*], Martina Hrast[a*]

[a] University of Ljubljana, Faculty of Pharmacy, Aškerčeva 7, SI-1000 Ljubljana, Slovenia

Table of contents

Table S1..2

Table S2..5

Table S3..7

Dose response curves..9

Table S1. *In vitro* inhibitory potencies of Merck's Mini Library against MurA, MurC, DdlB, mAChE, hBChE, hMAO-A/B.

Compound	hBChE (RA [%][a]/IC_{50})	mAChE (RA [%][a]/IC_{50})	hMAO-A (RA[a] [%])	hMAO-B (RA[a] [%])	MurC (RA[b] [%])	MurA (RA[b] [%])	DdlB (RA[b] [%])
**MS-ML10**	**35.6 ± 0.3 nM**	64	75	82	74	110	96
**MS-ML11**	**78.8 ± 10.3 nM**	82	31	45	89	100	95
**MS-ML12**	**4654 ± 712 nM**	108	75	81	88	100	96
**MS-ML13**	100	114	79	82	100	96	96
**MS-ML14**	93	110	84	87	105	100	97
**MS-ML60**	92	114	85	89	92	100	96
**MS-ML61**	81	109	96	96	101	105	105
**MS-ML62**	99	99	99	94	105	111	107
**MS-ML35**	105	102	92	89	104	101	103
**MS-ML36**	98	97	89	88	103	121	101
**MS-ML38**	96	88	84	89	102	100	101
**MS-ML37**	104	102	82	87	108	100	102
**MS-ML39**	101	93	84	88	101	100	103
**MS-ML40**	96	106	87	88	99	98	104
**MS-ML63**	92	99	86	92	86	100	105
**MS-ML64**	96	99	92	92	86	112	106
**MS-ML46**	104	99	100	93	102	104	103
**MS-ML47**	102	96	95	90	101	104	100
**MS-ML48**	103	98	87	89	102	66	104
**MS-ML53**	56	100	78	86	101	105	109
**MS-ML50**	92	98	61	87	104	79	104
**MS-ML49**	101	101	65	87	96	105	105
**MS-ML51**	**1594 ± 99 nM**	95	93	89	92	104	105
**MS-ML52**	90	94	100	87	93	99	112
**MS-ML57**	100	101	99	95	100	102	100
**MS-ML58**	101	88	96	89	98	98	98
**MS-ML59**	88	91	95	89	98	92	103

**MS-ML54**	103	111	86	90	99	102	99
**MS-ML55**	100	107	81	89	100	102	101
**MS-ML56**	97	106	42	85	99	85	100
**MS-ML22**	73	103	77	71	97	72	100
**MS-ML23**	73	99	90	90	103	103	113
**MS-ML06**	95	106	92	100	104	96	98
**MS-ML07**	91	98	91	95	99	96	100
**MS-ML08**	76	90	60	76	108	83	100
**MS-ML09**	92	88	86	92	89	94	91
**MS-ML41**	74	96	80	91	103	96	95
**MS-ML42**	102	105	85	92	100	100	96
**MS-ML43**	86	103	93	93	92	102	98
**MS-ML44**	101	97	94	98	95	109	110
**MS-ML01**	83	106	84	105	**45**	86	84
**MS-ML05**	91	96	79	93	**55**	90	91
**MS-ML03**	87	92	83	97	**61**	100	81
**MS-ML02**	103	110	73	94	74	106	90
**MS-ML04**	94	103	88	97	85	97	91
**MS-ML66**	101	102	67	100	72	79	81
**MS-ML65**	91	91	74	91	64	86	69
**MS-ML67**	103	98	77	94	76	98	106
**MS-ML16**	100	103	81	75	90	99	97
**MS-ML17**	99	112	72	85	100	94	103
**MS-ML18**	90	97	74	80	98	98	107
**MS-ML19**	97	107	49	72	100	98	102
**MS-ML69**	97	76	83	73	87	90	95
**MS-ML68**	28	66	96	86	94	85	90
**MS-ML72**	94	99	92	96	92	92	98
**MS-ML71**	102	99	74	89	92	92	102
**MS-ML70**	110	117	94	52	88	99	100

**MS-ML20**	100	117	94	91	92	99	101
**MS-ML21**	100	105	86	85	93	102	106
**MS-ML24**	60	**414 ± 40 nM**	57	64	83	**15**	116
**MS-ML25**	108	**369 ± 55 nM**	73	77	98	**0**	114
**MS-ML26**	68	**359 ± 39 nM**	60	92	73	**7**	114
**MS-ML33**	100	110	68	91	82	95	111
**MS-ML34**	106	102	78	79	96	130	99
**MS-ML27**	97	106	102	91	101	98	96
**MS-ML28**	102	103	99	89	92	89	100
**MS-ML29**	94	81	92	86	98	88	95
**MS-ML30**	85	104	79	80	90	89	94
**MS-ML45**	100	92	76	87	97	102	110
**MS-ML31**	84	89	78	**13**	94	94	98
**MS-ML32**	93	87	90	93	101	97	101
**MS-ML15**	103	108	94	96	89	110	97
**MS-ML73**	60	105	88	74	89	106	90
**MS-ML74**	84	103	86	83	103	101	93
**MS-ML75**	76	90	94	68	87	101	90
**MS-ML76**	61	107	78	81	86	105	98
**MS-ML77**	94	116	81	82	80	94	96
**MS-ML78**	97	104	82	81	98	98	99
**MS-ML79**	103	98	86	94	97	99	93
**MS-ML80**	73	95	82	**11**	96	93	94

[a] RA was determined at 10 μM; [b] RA was determined at 50 μM, the active compounds are bolded.

Table S2. Structures of salicylic acid analogues.

Compounds	Structure
**MS-ML24**	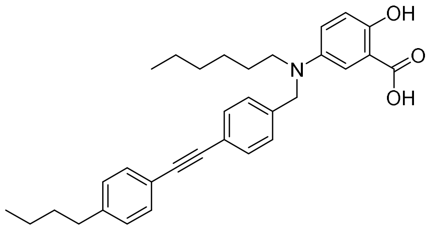
**MS-ML25**	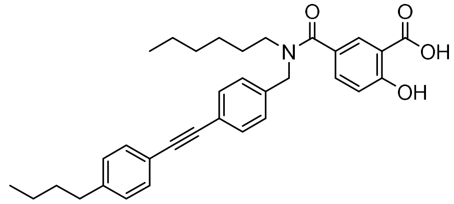
**MS-ML26**	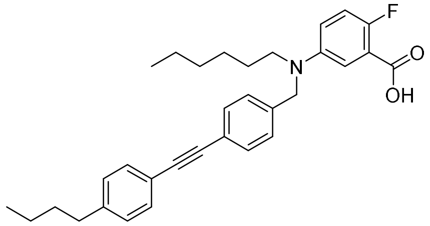
**1**	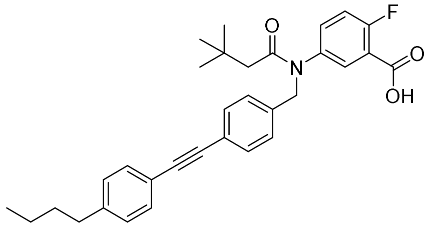
**2**	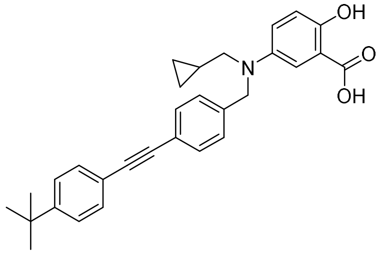
**3**	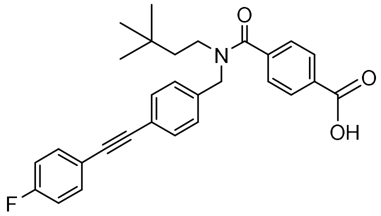
**4**	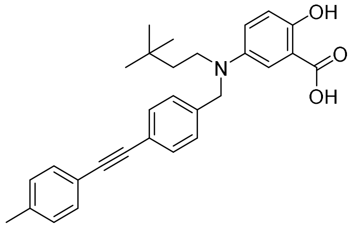

5

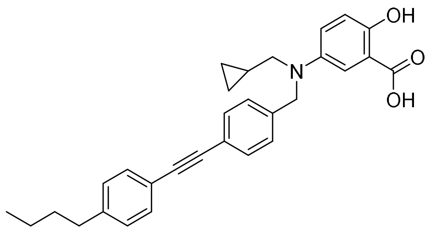

6

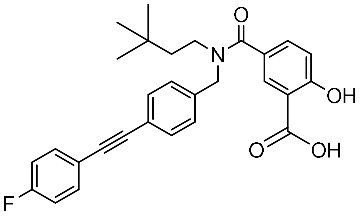

7

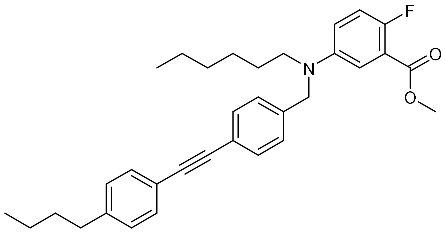

8

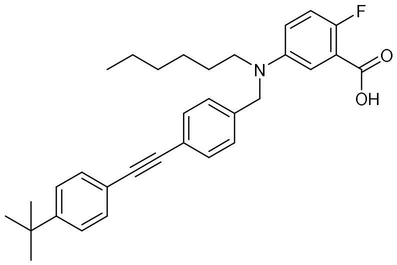

9

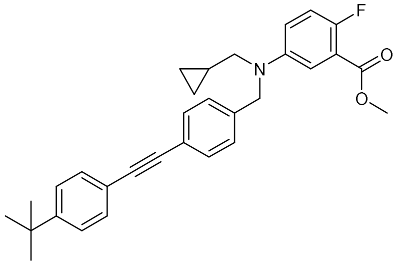

Table S3. *In vitro* inhibitory potencies of triazaindolizines derivatives against hMAO-B and hMAO-A.

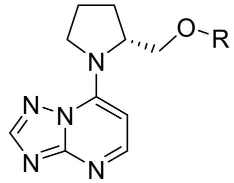

Compounds (Rack Barcode: DTBR00058180)	R	hMAO-B		hMAO-A
		RA[a] [%]	IC_{50} [nM]	RA[a] [%]
**MS-ML31**	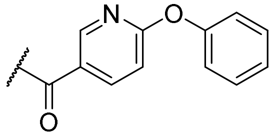	17	900 ± 100	98
**S1**	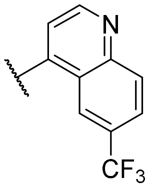	75	/	70
**S2**	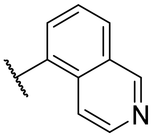	88	/	87
**S3**	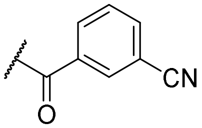	91	/	96
**S4**	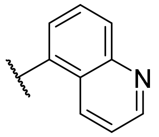	99	/	80
**S5**	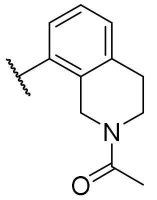	98	/	94
**S6**	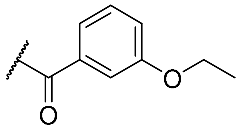	88	/	96
**S7**	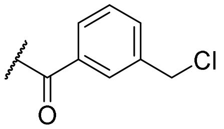	93	/	70

**S8**	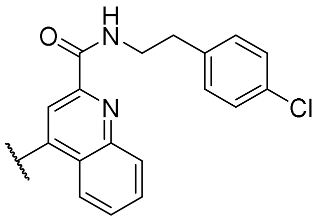	104	/	86
**S9**	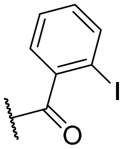	93	/	90
**S10**	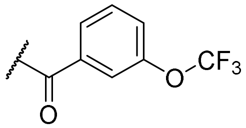	81	/	92

[a] RA was determined at 10 μM.

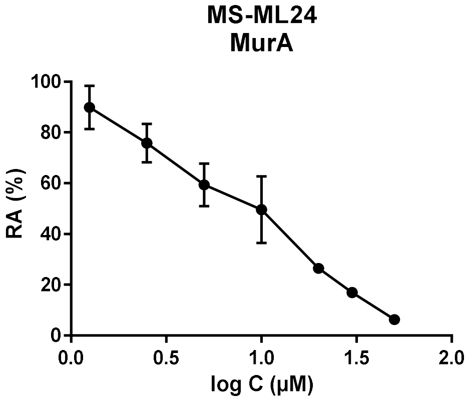
MS-ML24
MurA
RA (%)
log C (µM)

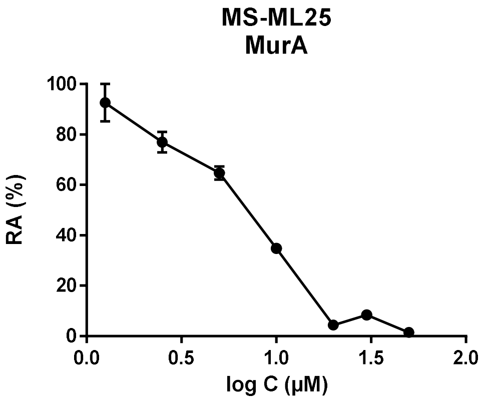
MS-ML25
MurA
RA (%)
log C (µM)

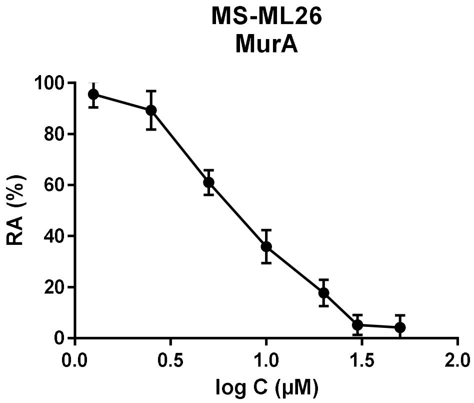
MS-ML26
MurA
RA (%)
log C (µM)

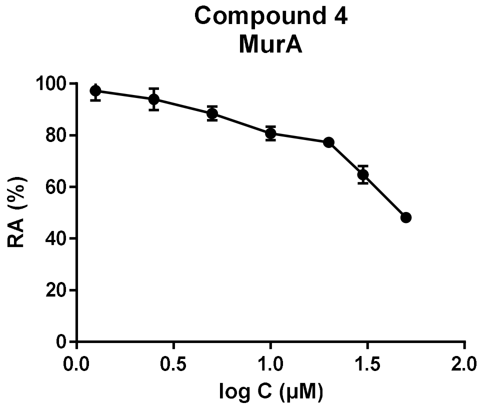
Compound 4
MurA
RA (%)
log C (µM)

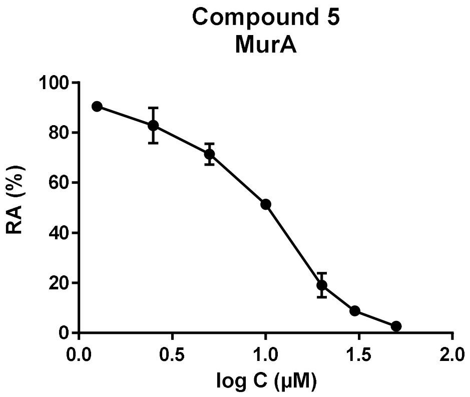
Compound 5
MurA
RA (%)
log C (µM)

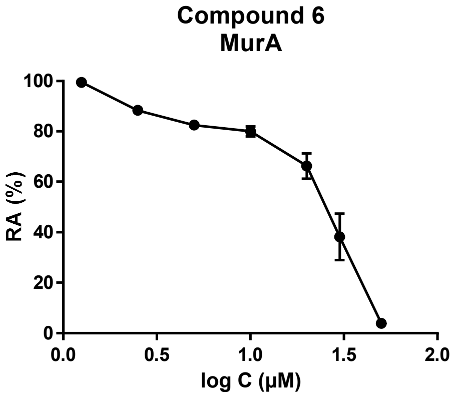
Compound 6
MurA
RA (%)
log C (µM)

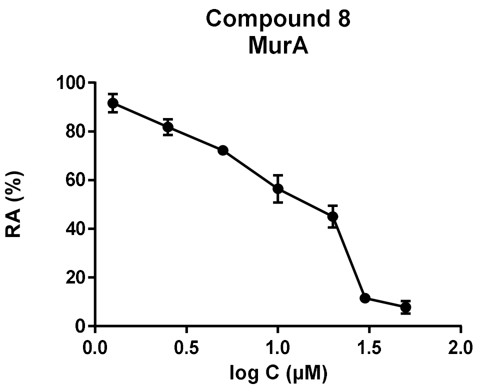
Compound 8
MurA
RA (%)
100
80
60
40
20
0
0.0
0.5
1.0
1.5
2.0
log C (μM)

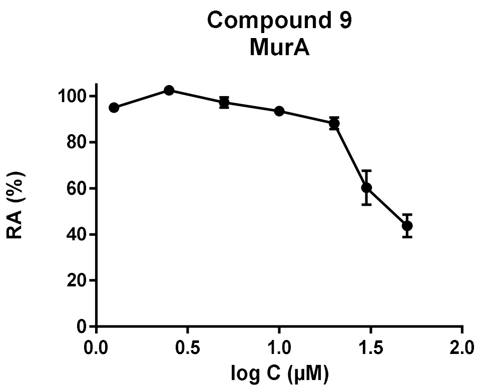
Compound 9
MurA
RA (%)
100
80
60
40
20
0
0.0
0.5
1.0
1.5
2.0
log C (μM)

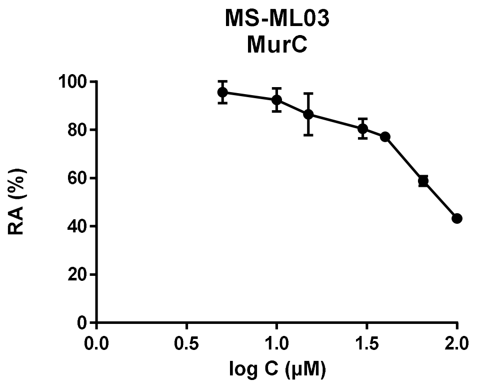
MS-ML03
MurC
RA (%)
log C (μM)
0
20
40
60
80
100
0.0
0.5
1.0
1.5
2.0

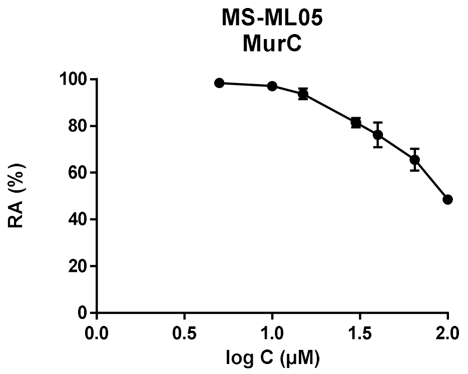
MS-ML05
MurC
RA (%)
log C (μM)
0
20
40
60
80
100
0.0
0.5
1.0
1.5
2.0

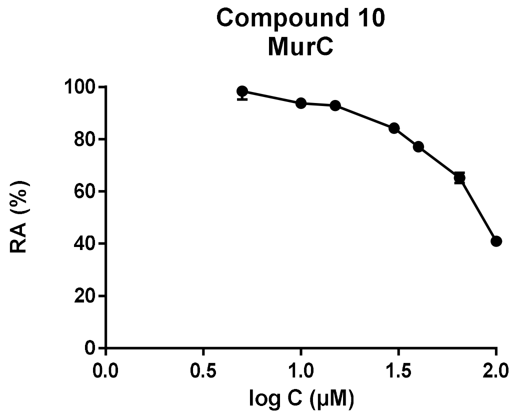
Compound 10
MurC
RA (%)
log C (μM)
0
20
40
60
80
100
0.0
0.5
1.0
1.5
2.0

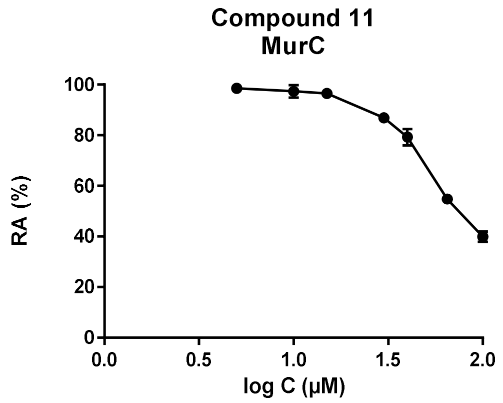
Compound 11
MurC
RA (%)
log C (μM)
0
20
40
60
80
100
0.0
0.5
1.0
1.5
2.0

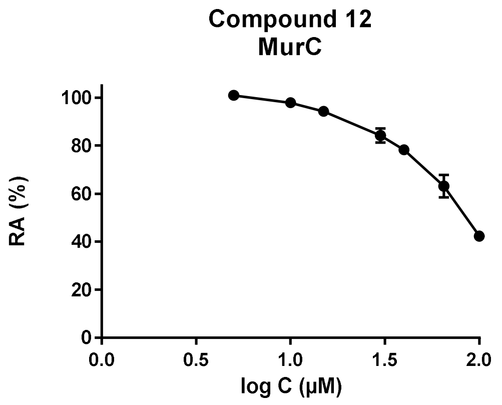
Compound 12
MurC
RA (%)
log C (μM)
0
20
40
60
80
100
0.0
0.5
1.0
1.5
2.0

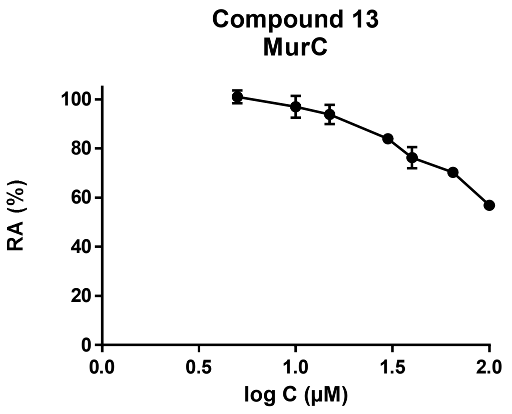
Compound 13
MurC
RA (%)
log C (μM)
0
20
40
60
80
100
0.0
0.5
1.0
1.5
2.0

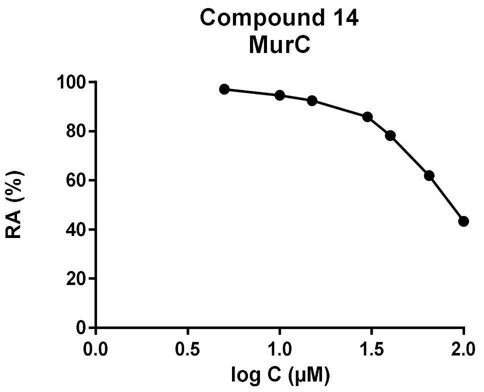
Compound 14
MurC
RA (%)
100
80
60
40
20
0
0.0
0.5
1.0
1.5
2.0
log C (μM)

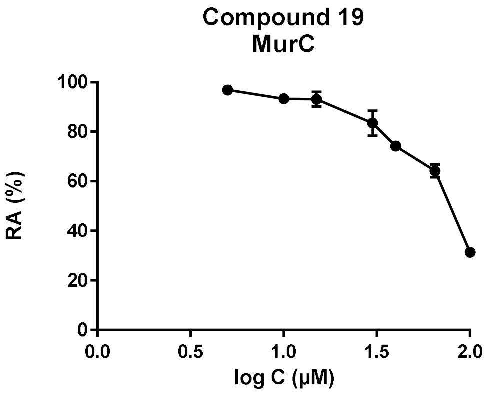
Compound 19
MurC
RA (%)
100
80
60
40
20
0
0.0
0.5
1.0
1.5
2.0
log C (μM)

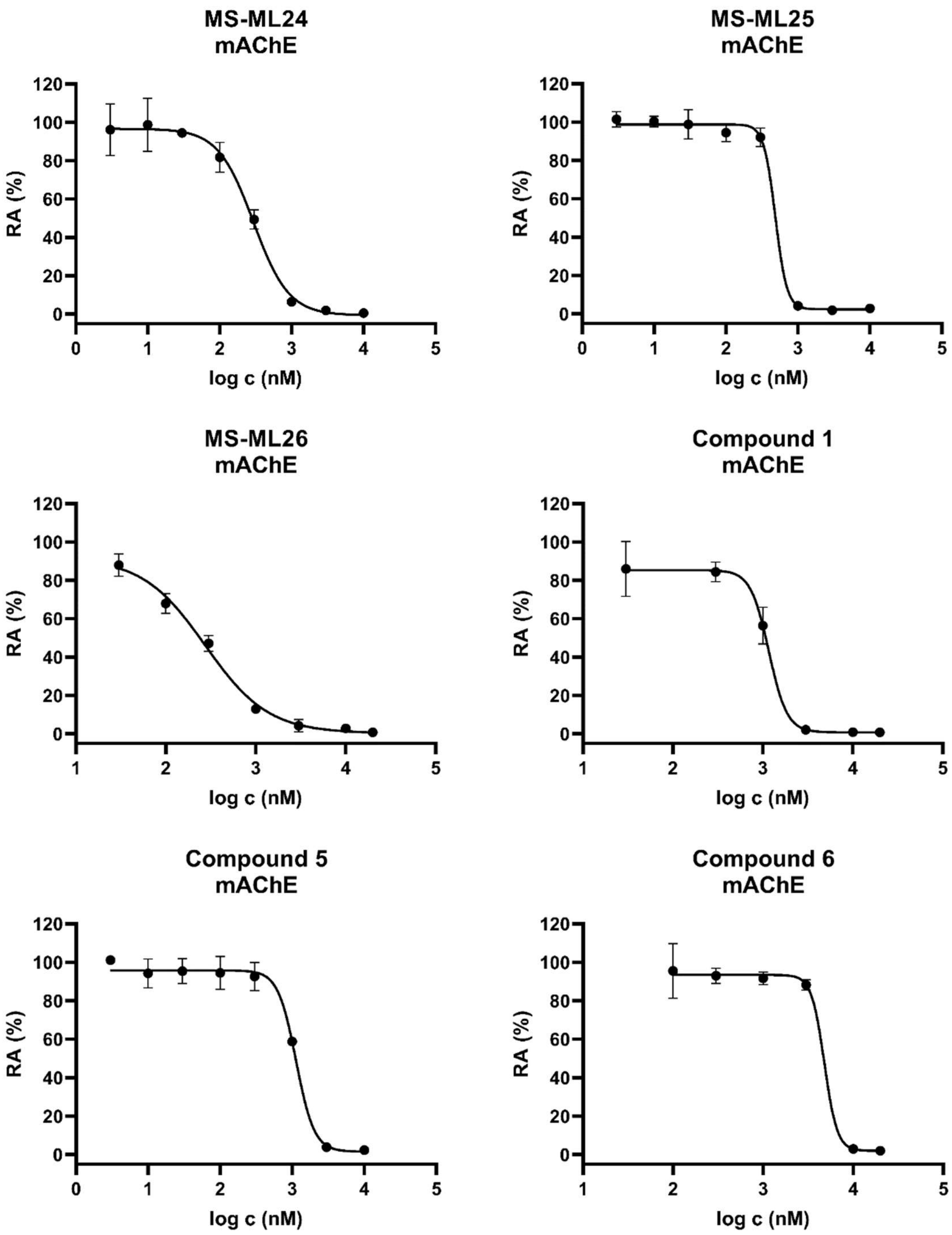
MS-ML24
mAChE
MS-ML25
mAChE
MS-ML26
mAChE
Compound 1
mAChE
Compound 5
mAChE
Compound 6
mAChE
RA (%)
log c (nM)
0
20
40
60
80
100
120
1
2
3
4
5

Compound 7
mAChE

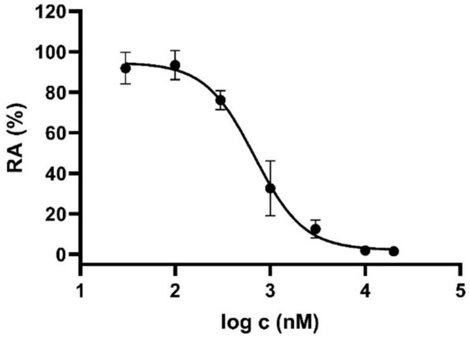

Compound 8
mAChE

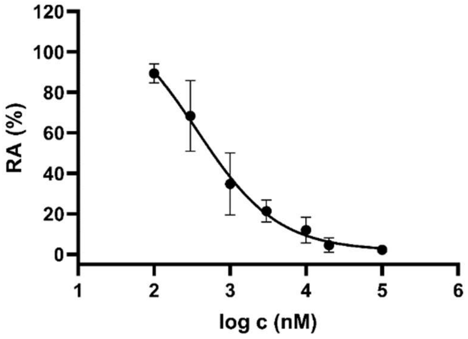

Compound 9
mAChE

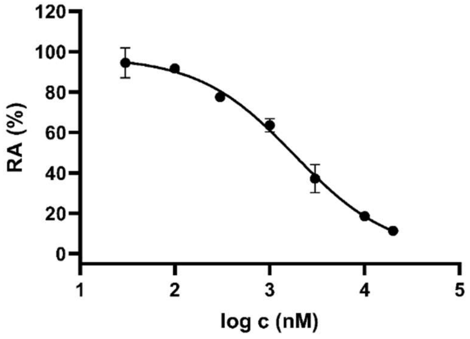

Compound 31
mAChE

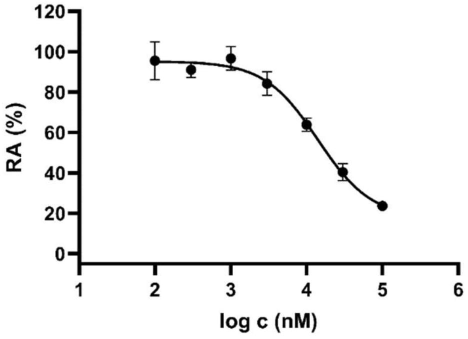

Compound 34
mAChE

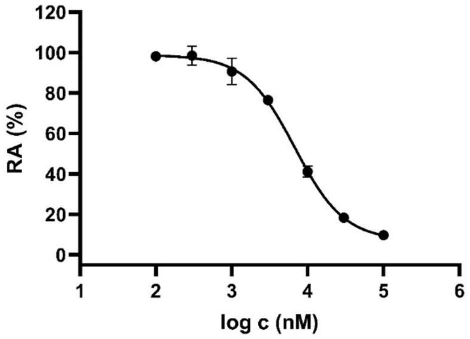

Compound 39
mAChE

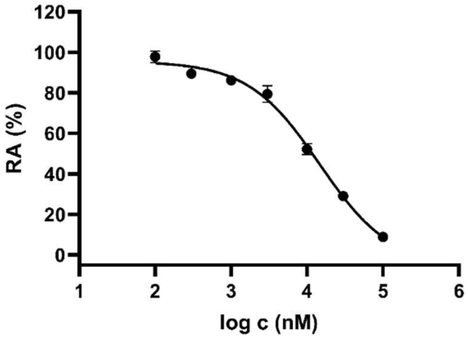

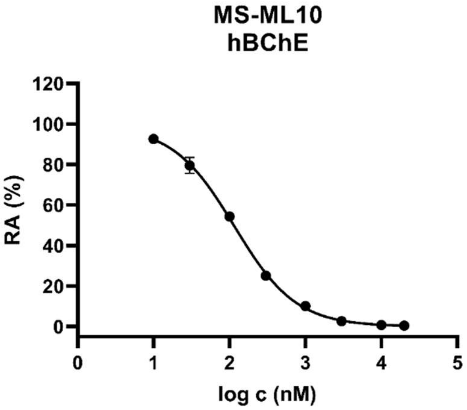
MS-ML10
hBChE
RA (%)
log c (nM)

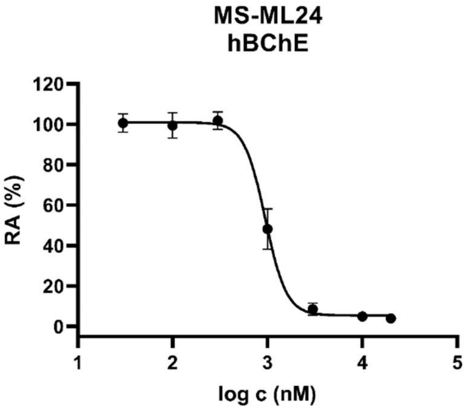
MS-ML24
hBChE
RA (%)
log c (nM)

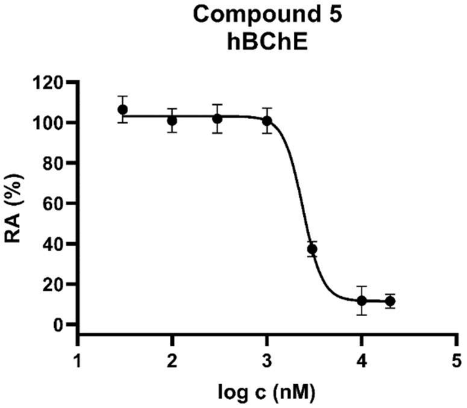
Compound 5
hBChE
RA (%)
log c (nM)

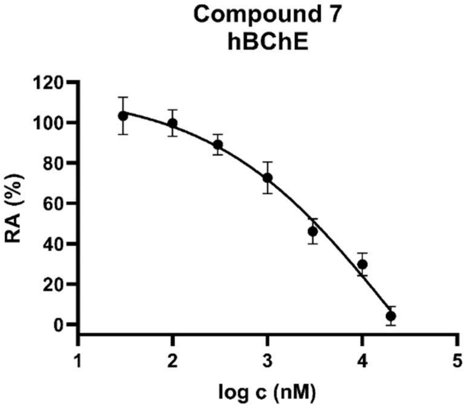
Compound 7
hBChE
RA (%)
log c (nM)

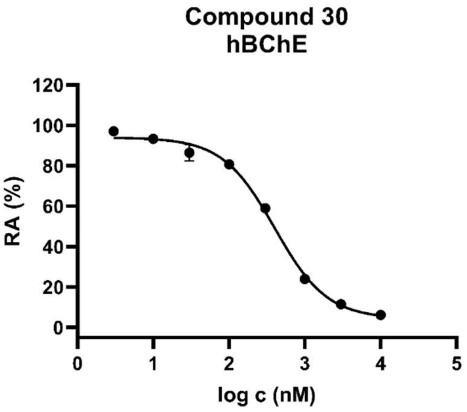
Compound 30
hBChE
RA (%)
log c (nM)

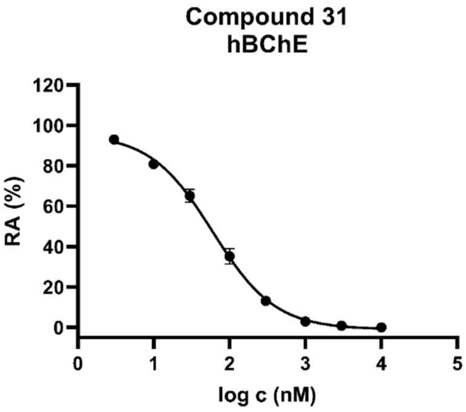
Compound 31
hBChE
RA (%)
log c (nM)

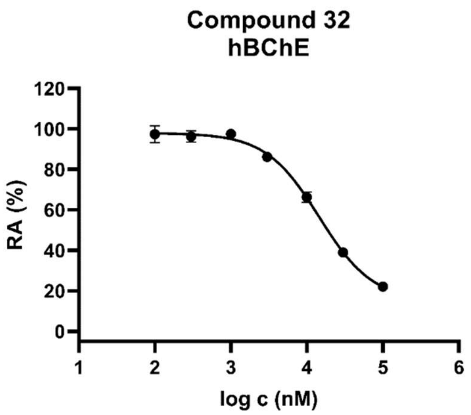
Compound 32
hBChE
RA (%)
log c (nM)

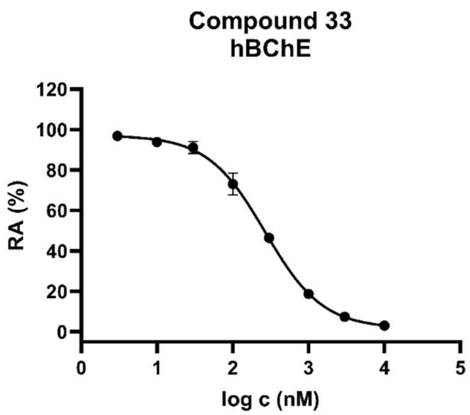
Compound 33
hBChE
RA (%)
log c (nM)

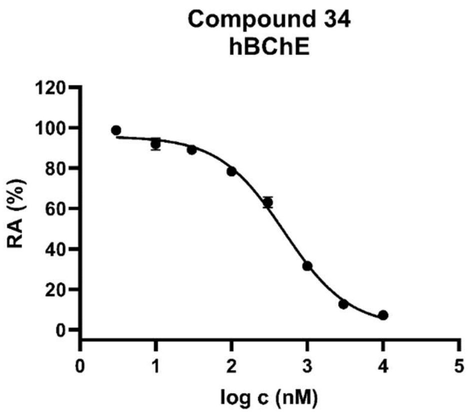
Compound 34
hBChE
RA (%)
log c (nM)

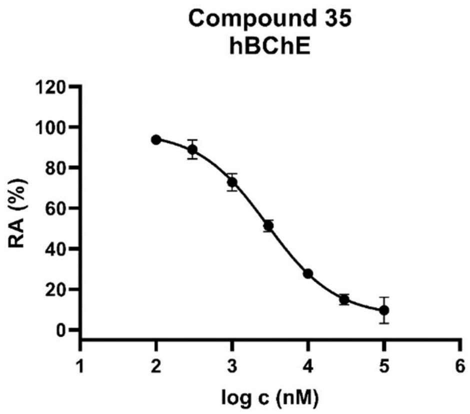
Compound 35
hBChE
RA (%)
log c (nM)

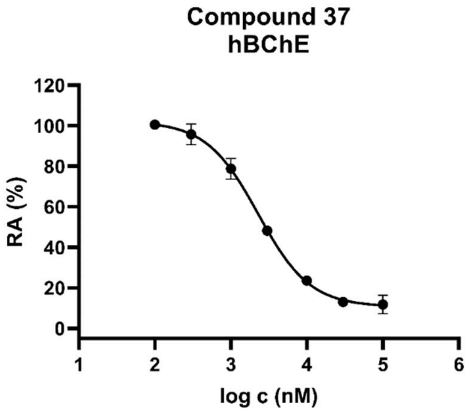
Compound 37
hBChE
RA (%)
log c (nM)

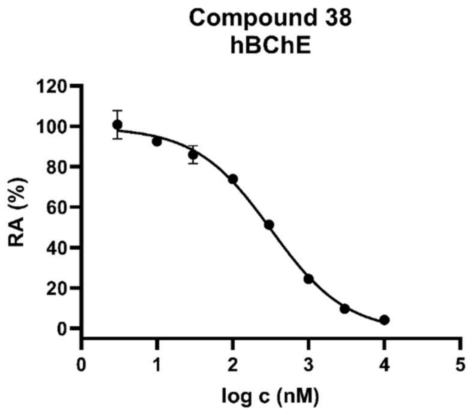
Compound 38
hBChE
RA (%)
log c (nM)

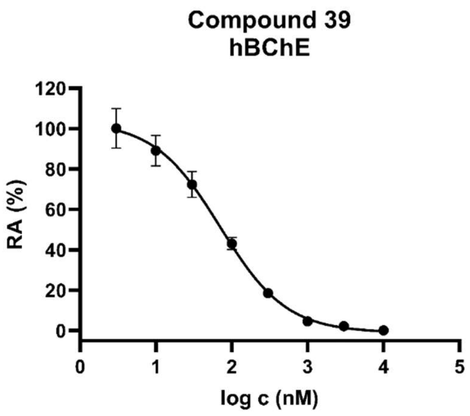
Compound 39
hBChE
RA (%)
log c (nM)
0
20
40
60
80
100
120
0
1
2
3
4
5

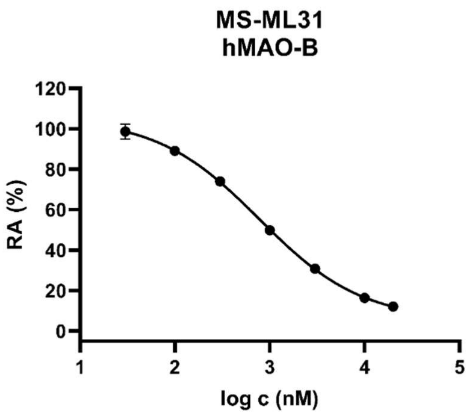
MS-ML31
hMAO-B
RA (%)
120
100
80
60
40
20
0
1
2
3
4
5
log c (nM)

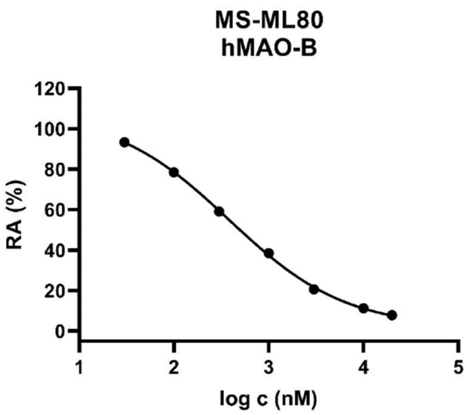
MS-ML80
hMAO-B
RA (%)
120
100
80
60
40
20
0
1
2
3
4
5
log c (nM)

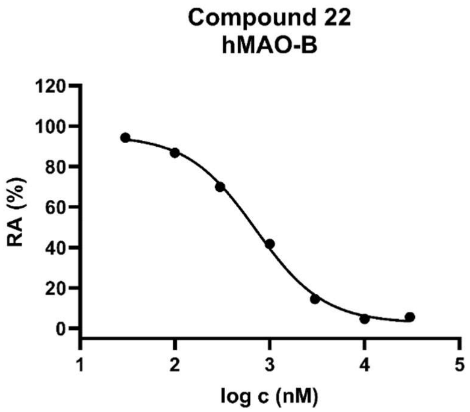
Compound 22
hMAO-B
RA (%)
120
100
80
60
40
20
0
1
2
3
4
5
log c (nM)

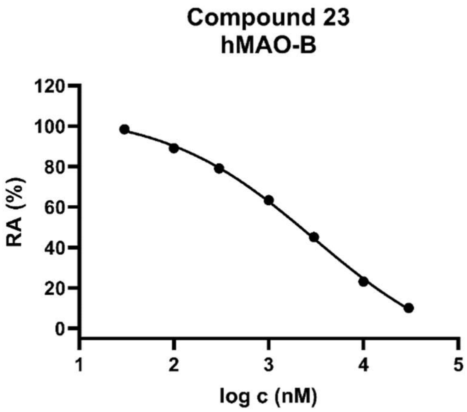
Compound 23
hMAO-B
RA (%)
120
100
80
60
40
20
0
1
2
3
4
5
log c (nM)

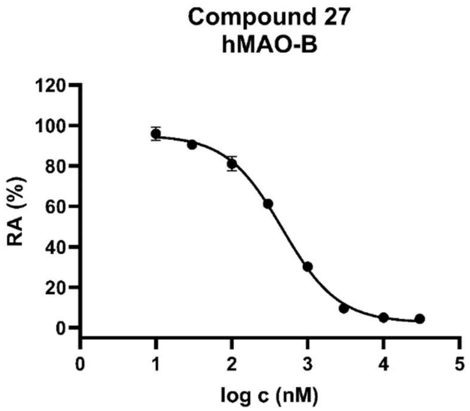
Compound 27
hMAO-B
RA (%)
120
100
80
60
40
20
0
0
1
2
3
4
5
log c (nM)

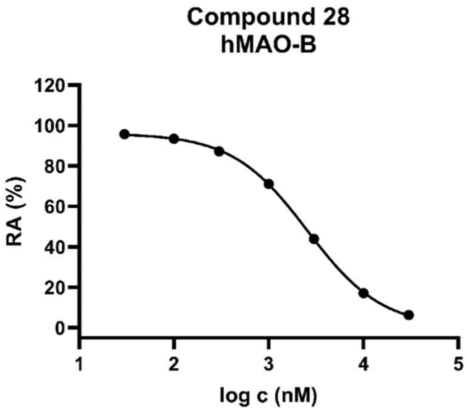
Compound 28
hMAO-B
RA (%)
120
100
80
60
40
20
0
1
2
3
4
5
log c (nM)
